# The Ninth International Congress

**Published:** 1885-10

**Authors:** 


### THE NINTH INTERNATIONAL MEDICAL CONGRESS.

The Ninth International Medical Congress will be held in Washington, D. C., United States of America, in August, 1887.

The committee of arrangements have done their duty admirably, and in the face of many trying difficulties, and the most annoying efforts to obstruct its work. Indifferent alike to criticism and cavil, these gentlemen have fearlessly discharged the responsible task of organizing the Congress and of arranging for the scientific working of the several sections, by the selection of men eminent in connection with the several branches of medical study. They, having finished their labors, pass off the stage now, and all further details will be attended to by the Executive Committee. The committee, however, will not be formally dismissed until after its report is made to the American Medical Association at St. Louis next April. They deserve the thanks of the Association, and of all true physicians and lovers of medical science.

We regret we have not the space to reproduce the rules, and to give a list of the officers. They can be found in the Journal of the Association.

Now, all members of the medical profession of America who desire the good of the profession and the advancement of medical science above any mere personal gratification of ambition, are called upon and expected to co-operate heartily in the work of making the Congress successful and creditable to America. Any further obstruction, or attempt at obstruction, will surely receive the contempt which it deserves at the hands of all civilized people. Fall into line, now, and go to work with a good will.

